# Critical Environmental Factors for Transportation Cycling in Children: A Qualitative Study Using Bike-Along Interviews

**DOI:** 10.1371/journal.pone.0106696

**Published:** 2014-09-24

**Authors:** Ariane Ghekiere, Jelle Van Cauwenberg, Bas de Geus, Peter Clarys, Greet Cardon, Jo Salmon, Ilse De Bourdeaudhuij, Benedicte Deforche

**Affiliations:** 1 Department of Human Biometry and Biomechanics, Faculty of Physical Education and Physical Therapy, Vrije Universiteit Brussel, Brussels, Belgium; 2 Department of Movement and Sport Sciences, Faculty of Medicine and Health Sciences, Ghent University, Ghent, Belgium; 3 Fund for Scientific Research Flanders (FWO), Brussels, Belgium; 4 Department of Human Physiology, Faculty of Physical Education and Physical Therapy, Vrije Universiteit Brussel, Brussels, Belgium; 5 Centre for Physical Activity and Nutrition Research, School of Exercise and Nutrition Science, Deakin University, Melbourne, Australia; University of Tolima, Colombia

## Abstract

**Background:**

Environmental factors are found to influence transport-related physical activity, but have rarely been studied in relation with cycling for transport to various destinations in 10–12 yr old children. The current qualitative study used ‘bike-along interviews’ with children and parents to allow discussion of detailed environmental factors that may influence children's cycling for transport, while cycling in the participant's neighborhood.

**Methods:**

Purposeful convenience sampling was used to recruit 35 children and one of their parents residing in (semi-) urban areas. Bike-along interviews were conducted to and from a randomly chosen destination (e.g. library) within a 15 minutes' cycle trip in the participant's neighborhood. Participants wore a GoPro camera to objectively assess environmental elements, which were subsequently discussed with participants. Content analysis and arising themes were derived using a grounded theory approach.

**Results:**

The discussed environmental factors were categorized under traffic, urban design, cycling facilities, road design, facilities at destination, aesthetics, topography, weather, social control, stranger danger and familiar environment. Across these categories many environmental factors were (in)directly linked to road safety. This was illustrated by detailed discussions of the children's visibility, familiarity with specific traffic situations, and degree of separation, width and legibility of cycle facilities.

**Conclusion:**

Road safety is of major concern in this 10–12 yr old study population. Bike-along interviews were able to identify new, detailed and context-specific physical environmental factors which could inform policy makers to promote children's cycling for transport. However, future studies should investigate whether hypothetical changes to such micro environmental features influence perceptions of safety and if this in turn could lead to changes in children's cycling for transport.

## Background

Many children around the world do not meet the recommended amount of physical activity [1], [2]. Promoting active transport (i.e. walking or cycling to a specific destination) is considered one solution to integrate physical activity into children's daily life [3], [4]. Walking and cycling are inexpensive and very accessible forms of physical activity. In order to design effective interventions to promote walking and cycling, there is a need for studies identifying its influencing factors [5]. Cycling for transport has been linked to higher total physical activity levels [6], [7], better cardiorespiratory fitness [8], [9], and a healthier body composition [10] in children. Cycling has also some ecological and economic benefits, e.g. cycling can also reduce noise, traffic congestions, air pollution and carbon emissions [11]. Despite these advantages, cycling is the main transport mode in only 11% of Flemish 6- to 12-year old children [12]. Knowing that independent mobility increases from the age of ten and physical activity levels of children decrease during transition into adolescence [13], children in their last years of primary school are an important target group to encourage cycling for transport [14].

Ecological models differentiate multiple levels of influence on cycling for transport [15]. The environment, defined by World Health Organization (WHO) as the physical, natural and social context in which the individual spends his or her time [16], is known to be one of these influencing factors [17], [18], [19], [20]. A systematic review by Fraser and Lock (2011) identified different environmental factors related to cycling for transport across different age groups [21]. Only seven studies investigated environmental correlates of cycling in primary school children and all of them were conducted in the United States, Canada or Australia. Furthermore, in six of the seven studies, walking and cycling were combined into a single active transport behaviour, even though it has been recommended that walking and cycling should be examined as separate transport modes [22]. More recently, three other studies determined the relation between the physical environment and cycling for transport in 9- to 12- year old children [23], [24], [25]. These studies only focused on cycling for transport to school and did not include cycling to various destinations. However, cycling to destinations other than school is also of interest since children may cycle locally to friends, family or leisure activities [26] and the physical environment is likely to play an important role in whether children visit these destinations.

Cycling on busy roads, having to cross many roads, high traffic density, parental concern about stranger danger and having no safe place to cross are negatively related to cycling for transport in children [23], [25], [27], [28], [29], [30]. Positive associations of the presence of recreation facilities, cycle store facilities, pedestrian crossings, cycling along a quiet route, walkway quality, and walkability are identified [23], [25], [27], [28], [29], [30]. The presence of green space, water, cycle tracks, traffic lights, roundabouts and intersections, traffic safety, connectivity, hilliness, residential density, and width of cycle lanes are elements of which the association with cycling for transport in children is inconclusive [23], [25], [27], [28], [29], [31].

Parents play a key role regarding whether or not their child cycles for transport independently of a supervising adult [15], [32], [33], [34]. The ultimate decision on transport mode in this age group is provided by mutual discussion between parent and child [15]. Starting from the age of ten, children are asking more for independent mobility, implying that their willingness to cycle is also important in the decision process [35]. Therefore, studies using perceptions of both parents and their children are needed, as often only perceptions of parents are studied.

Most previous studies have used surveys to assess perceived cycling friendliness of an environment. Surveys require an intellectual capacity of the participants to remember the environmental characteristics of a cycling environment while not being in this target environment. To study more context-specific and detailed environmental elements, ‘go-along’ methods have been proposed as an approach that could be used for improving the understanding of people's experiences while being in the target-environment [36], [37]. A previous study using walk-along interviews in older adults elicited very rich information on micro-scale environmental factors (e.g. quality of sidewalks, openness etc.) that were perceived to influence walking behaviour [38]. It can therefore be expected that “bike-along” interviews can reveal context-specific and detailed environmental elements related to cycling for transport in children which cannot be captured by surveys. Furthermore, these bike-along interviews may establish insights regarding how and why these elements are related to cycling for transport in children within their neighbourhood [39].

The current study will be the first to use bike-along interviews with children and their parents. The aim was to identify context-sensitive environmental factors facilitating or hindering cycling for transport to multiple destinations in 10- to 12-year old children.

## Methods

### Participants

A sample of 35 children (10–12 years of age) and one of their parents was recruited by face-to-face contact or by telephone via purposeful convenience sampling, until theoretical saturation. The aim was to include both regular and non-regular cyclists and both boys and girls. In order to provide sufficient variation in environmental settings, children residing in different municipalities across Flanders were sought. Participants resided in either an urban (>600 inh./km^2^) or semi-urban (300–600 inh./km^2^) area [40]. Flanders is the Northern part of Belgium, has 462 inhabitants/km^2^ and is characterized with a mild sea climate, a flat landscape, a dense network of cycle paths and a high residential density [26], [41]. In semi-urban areas, the residential density is lower than within the city centers. After Denmark and The Netherlands, Flanders has the highest share of transportation cycling across the world [42], but still many children are chauffeured to destinations, as the car remains responsible for most trips (64.4%) among children [12]. For children, cycling remains the most dangerous transport mode to get to a destination, with a much higher risk of being (seriously) injured or killed compared as being a passenger in the car [43]. Additionally, most Flemish households have at least one car available [26].

The study protocol was approved by the ethical committee of the University Hospital of the Vrije Universiteit Brussel. All children and one of their parents provided written informed consent and gave permission for using their de-identified quotes in research publications.

### Procedure and measures

Participants were visited at home by two trained female researchers, one PhD and one master degree student, to complete the study procedure consisting of three consecutive parts: (1) a questionnaire concerning demographic characteristics and physical activity and active transport patterns; (2) a cycling trip to a destination in the participant's neighborhood; and (3) a semi-structured interview. Conducting these three parts of the study procedure took approximately two hours.

Each parent and child was asked to complete a questionnaire. The children's questionnaire asked about their age and sex and they had to rank five different transport modes (on foot, by bike, car, public transport or skateboard/inline skates/step) according to preference for going to school and to other destinations. The parental questionnaire determined several demographic characteristics (e.g. age, sex, place of residence, educational level, main occupation). Physical activity and active transport patterns were questioned based on the validated Flemish Physical Activity Questionnaire [44] for child's behavior and the International Physical Activity Questionnaire (short form) [45] for parent's behavior. Parents reported their frequency and duration of walking and cycling in a usual week, as well as that of their child. Parents also reported their child's main transport mode to school. To determine independent mobility, parents were asked which distance (six point scale: not, 0–500 m, 500–1000 m, 1–3 km, 5–10 km, more than 10 km) their child was allowed to cycle alone. Distance to facilities in the participant's neighborhood was examined by using the “Stores, facilities and other things in your neighborhood” section of the Neighborhood Environment Walkability Scale (NEWS) [46]. This section was extended with destinations relevant to children (e.g. a playground, a friend's home, scouts). Based upon this last question, a destination within a 10–15 minutes' ride on the bike was randomly chosen for the bike-along interview.

After filling in the questionnaires (duration child: 10–15 minutes, parent: 20–30 minutes), the child and one of their parents made a cycling trip with the researcher to the randomly chosen destination (e.g. school, a friend's home, library, shop). In bike-along interviews, the researcher accompanies the participant while cycling in the participant's environment, so that the participant is able to discuss experiences, feelings or ideas while cycling through this area. Here, the interaction between the participants and their own environment offers the opportunity to explain which environmental elements they (dis)like, but also how and why they are important to them. This qualitative research method benefits from a unique interplay between the environment and participant but also the researcher who stimulates the participant to discuss the environmental factors.

The route to the destination was the route that participants mostly used when visiting this destination. The route back was cycled via another, less frequently used route chosen by the researcher to provide additional environmental variation. For safety reasons, the parent cycled in front, the child in the middle and followed by the researcher. Both parent and child wore a helmet with a sports camera (GoPro Hero2, outdoor edition). The use of GoPro cameras allows to record video (i.e. the encountered environment) and audio (i.e. corresponding comments of parent and child) during the cycle trip to the destination. Before starting the trip, the researcher explained the aim of the tour to both the parent and child.

“We will now cycle to ‘destination X’. The purpose is that you tell me which environmental elements make it more or less difficult, more or less enjoyable, comfortable or interesting to cycle in this environment. Consider also elements that affect your safety feelings. This may be related to road safety and safety from crime, but also safety of being injured. Thus, think about all the positive and negative things in the environment that influence how you experience your cycling trip. You are the expert and the purpose is that you freely inform me about your experiences, ideas and opinions so that I can learn about the things in the environment that facilitate or hinder your cycling. I might ask additional questions to understand completely your experiences, ideas and opinions. So you will ride in front of me and discuss which elements you find good or bad, fun or less fun, easy or less easy to cycle for transport. What you tell is recorded by the camera, so you do not have to target me when telling something, but it is important that you look in front of you, like how you would cycle alone. It is possible that during the cycle trip, there is no time to discuss all elements, but that is not a problem, because we can watch the video on the computer afterwards and you can further explain your experiences, ideas and opinions. Is everything clear?” Parents were similarly instructed to indicate which environmental elements had an impact on their willingness to let their child cycle alone in this environment.

The third part of the study consisted of discussing the video recordings. The video recordings were uploaded on the computer immediately after arriving at the participants' home. The video could then be viewed in a video media player (i.e. VLC Media Player). It did not need any editing, video and audio recordings were simultaneously presented to the participants. Children examined their own video recordings along with the researcher, separately from their parents who also revised their recordings with the researcher. Through an open discussion between child/parent and the researcher, ambiguities could be clarified and other environmental elements could be identified. This discussion was conducted immediately after returning at the participants' home. There was no specific interview structure that was followed by the researchers. Data collection was performed in various weather conditions (rain, sun, snow, cold, dry) by two trained researchers by daytime during the period February-May 2013.

### Data analysis

Data obtained via the questionnaires were used to calculate descriptive statistics in SPSS version 20. Data obtained via interviews were audio-taped and transcribed verbatim afterwards. Content analysis was performed using Nvivo 9 software (QRS International). Data analysis was guided by grounded theory, which is based on constructing factors through data analysis [47]. Two independent researchers carefully read the interviews and assigned factors to elements of the environment that were mentioned by the participants. These factors were subsequently combined in order to determine subcategories. The final categories were assessed by grouping these subcategories according to the terminology used in the WHO definition of environment [16]. In case of disagreement or doubts, a third researcher was consulted until consensus was reached. This determination of categories was conducted separately for children and parents, but one final schematic overview was created including both parental and children's correlates. Photographs were made from the videos which were, together with quotes from the participants, used to illustrate the findings.

## Results

### Sample characteristics

Descriptive characteristics of children and parents are presented in Table 1. Children's most frequently preferred transport mode to school and to other destinations was cycling (54% and 57% respectively), while the most frequent actual transport mode to school was motorised transport (43%). On average, children were allowed to cycle 3.2 km alone (range 0–10 km).

**Table 1:** Descriptive statistics of the sample (n = 35 children; n = 35 parents).

**Demographics**	
Child's age (M±SD)	11.2±0.5
Parent's age (M±SD)	42.0±4.5
Living in urban areas (%)	65.7
Regular cyclists (%)	60
Girls participating (%)	65.7
Mothers participating (%)	80
Parents being maried/cohabiting (%)	88.6
Parents having higher education (%)	74.3
Parents principal occupation (%)	
blue collar worker	37.1
white collar worker	51.4
no principal occupation	11.4
Numbers of cars in the household (%)	
0	2.9
1	42.9
≥2	54.3
**Transport behaviors (min/week) (M±SD)**	
Child's walking	50.0±50.3
Child's cycling	63.0±55.4
Parent's walking	41.3±63.2
Parent's cycling	99.5±132.4
**Child's prefered transport modes**	
***to school (%)***	
Cycling	54.3
Step/skate/skateboard	22.9
Walking	14.3
Car	8.6
Public transport	0
***to other destinations (%)***	
Cycling	57.1
Car	17.1
Walking	14.3
Step/skate/skateboard	8.6
Public transport	2.9
**Child's actual transport mode to school (%)**	
Motorised transport (car & public transport)	42.9
Cycling	34.3
Walking	22.9
**Child's independent mobility (M±SD)**	
meters allowed to cycle alone	3221±3372

M = mean; SD = standard deviation.

### Content analysis

**Figure 1:**
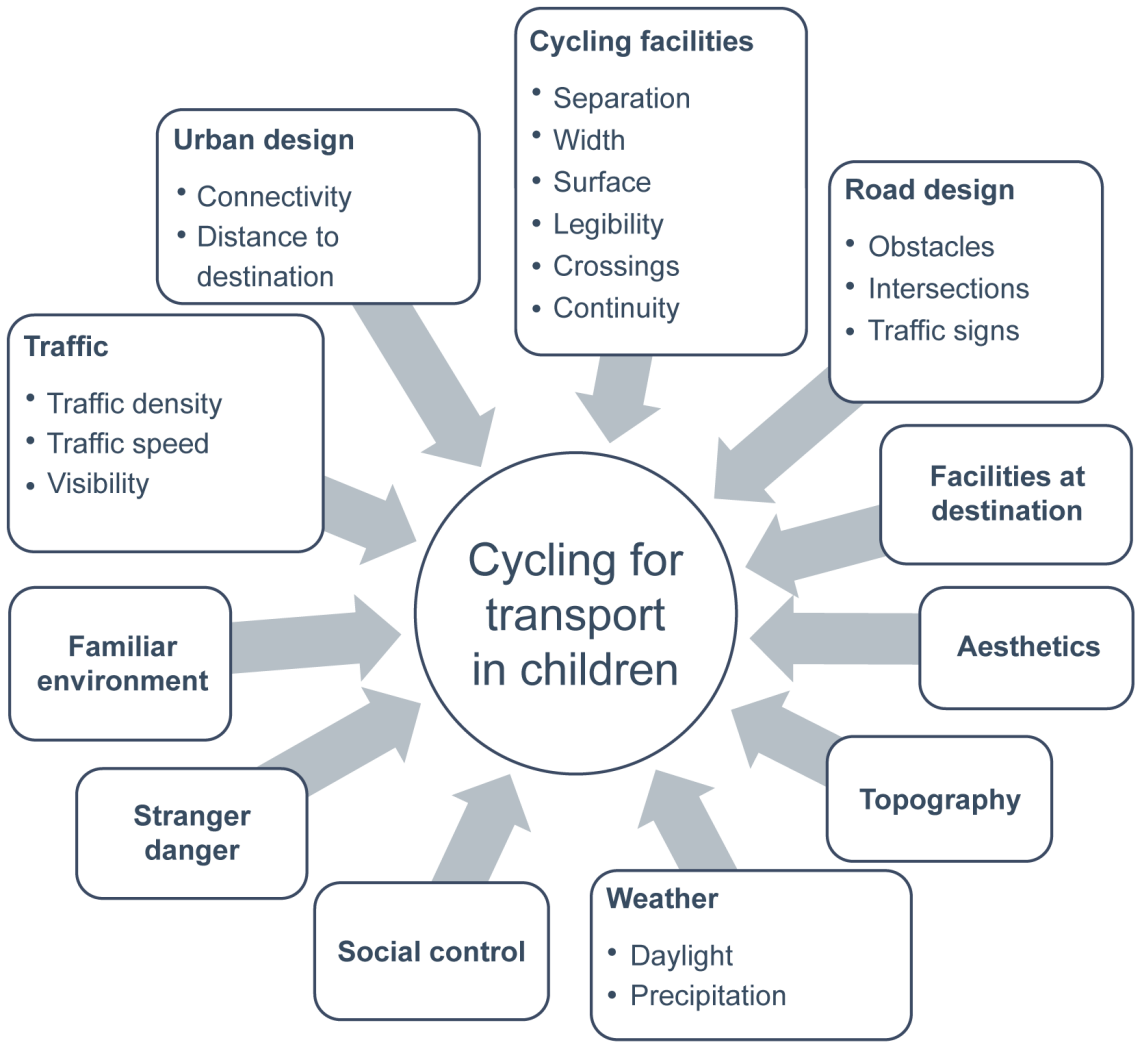
Environmental (sub-)categories identified by parents and children as potentially influencing children's cycling for transport.

Figure 1 shows the environmental (sub-)categories parents and children mentioned during the bike-along interviews. This structure will be used to represent the results. Three main categories were identified according to the WHO definition: the physical environment, the natural environment and the social environment. The physical environment included traffic, urban design, cycling facilities, road design and facilities at destination. The natural environment included aesthetics, topography and weather. Finally, the social environment included social control, stranger danger and familiar environment.

### Traffic

Traffic was extensively discussed by both parent and child in each bike-along interview. Parents indicated that traffic situations are a major concern for letting their child cycle for transport. Children also extensively discussed traffic, but they mentioned that other environmental elements were at least equally important to them.

#### Traffic density

Both parents and children indicated that it was more comfortable to cycle in streets with low traffic density. The presence of cars, busses, trams, trucks, pedestrians and other cyclists made it more difficult to have an overview of the road situation and, thus, made it less pleasant to cycle for children. Both parents and their children liked residential areas to cycle for transport since only local traffic passes through these areas.

#### Traffic speed

Parents and children agreed that streets with speed limitations were more inviting to cycle. They indicated that chicanes, speed bumps and speed limitations were helpful in slowing down the traffic. However, children also said that those chicanes are sometimes problematic since some cars cut corners and they drive very close to them:

*“A chicane, a bit difficult when cars need to pass. But it is made for cars in order that they drive slower and cars are actually driving slower here! ” (Girl, 10.2 yrs)*

#### Visibility

**Figure 2:**
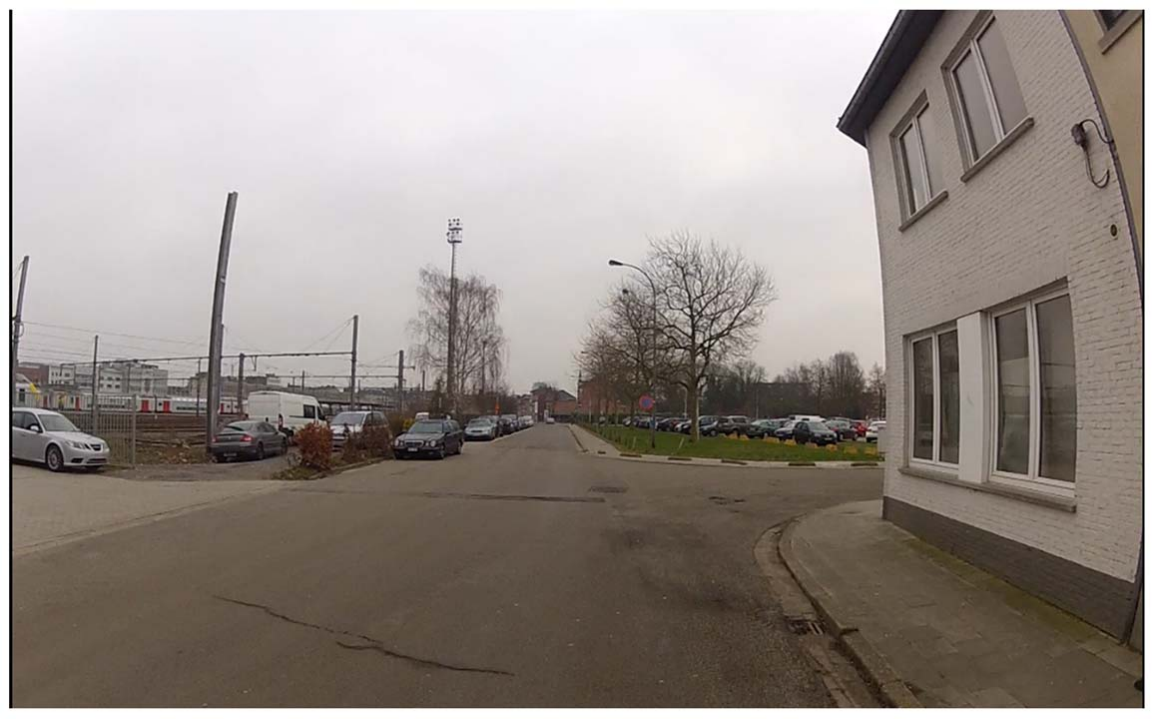
A situation where the view of oncoming traffic is impeded by the corner house.

Having a view of oncoming traffic, but also being visible themselves are two elements that were discussed by parents and children. Participants explained that the view of oncoming traffic could be impeded by buildings on street corners (see Figure 2) and curves in the streets. Trees or other natural elements and parked cars are at children's eye height and may reduce visibility. Wearing fluorescent clothing and lighting on the bicycle were seen as helpful tools to increase the visibility of children in traffic, which were, however, disliked by children.

### Urban design

#### Connectivity

To allow their child to cycle, parents indicated they liked having different routes to reach the destination, implying a preference for higher connectivity to cycle for transport. When several routes are present, parents prefer their child to cycle a bit longer but on good cycle paths than taking the shortest route on bad cycle paths.

*“To go to the store you can go along this road, but there are always alternatives to take, but then you have to make a detour. We always choose for the alternatives because that road is really not pleasant. The alternatives are, however, usually around 3 km further, but we do it anyway. Especially when my son will go to secondary school next year. I would prefer my son cycling 3km longer, so that he goes along paths that are somewhat separated from traffic, which is much safer …” (Father of boy, 40.1 yrs)*

#### Distance to destination

Children were more likely to cycle short distances, but when the destination was too short (e.g. less than five minutes walking), parents were more likely to let their child walk to the destination. Remarkably, parents often overestimated the time to reach the chosen destination and were often surprised that the destination was reached so quickly.

### Cycling facilities

#### Degree of separation

**Figure 3:**
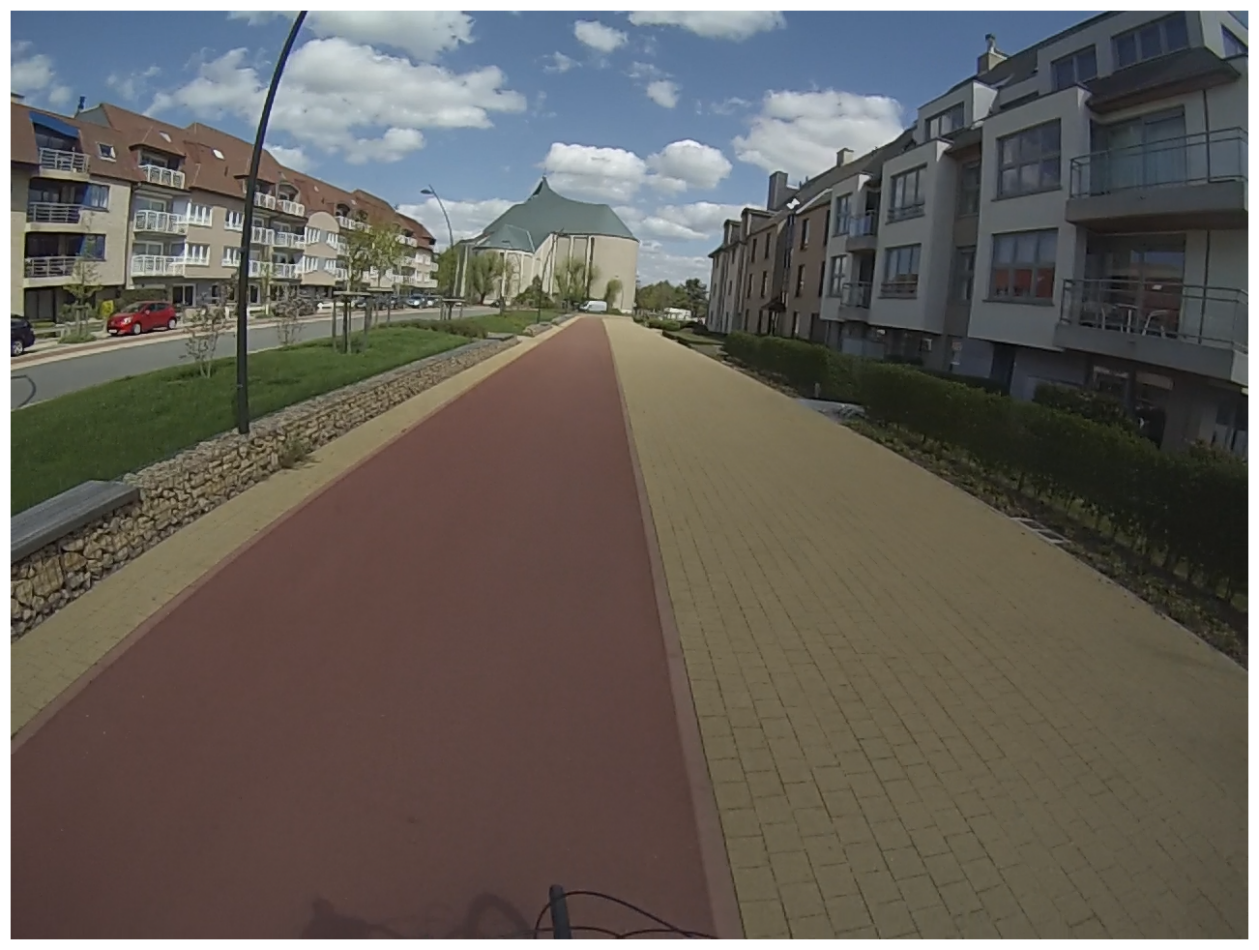
A good cycle path which is well separated from traffic, wide and without irregularities.

Children and parents felt more comfortable when there are cycle facilities, separated from the road by parked cars, a small hedge, a shoulder or when the cycle path was a bit higher than the road (see Figure 3). Parents were a bit critical about parked cars, because of the possibility of doors suddenly opening. Cycle lanes on the road were viewed less favorably than separated cycle tracks.

*“Here the cycle path is easy, beautiful and well-separated from the motorway. So it is quite safe to cycle with kids. The cycle path is fairly new and is very easy to use. We actually use it several times a day.” (Mother of a girl, 38.2 yrs)*

#### Width

Children and parents indicated that wider cycle paths were more enjoyable to cycle, so that children can cycle next to each other. Parents stated that it is especially important to have a wide cycle path, since children of that age may still have difficulties cycling on a straight line. When no cycle path was present, parents and children mentioned the importance of wide street lanes such that cars can easily pass. Also, bollards next to the cycle path or curbs of sidewalks were seen as making it difficult to move aside when cars need to pass.

*“I think oncoming cyclists are always difficult. … The cycle paths are just too narrow to cycle in two directions. If you cycle alone, then there is no problem, but I always fear that children are going to clash. Children will cycle more in the middle of the cycle path.” (Mother of girl, 40.7 yrs)*

#### Type of surface

**Figure 4:**
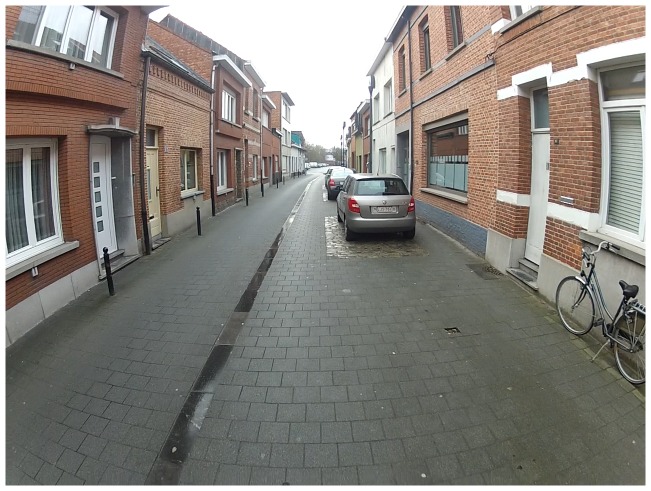
A slippery gutter in the middle of the road which was disliked by children.

Cobblestones, small pebbles or sand, but also tramways and manhole covers and uneven surfaces of the streets or cycle paths are elements that made it less comfortable and less appealing to cycle. Children were afraid of falling when cycling on certain surfaces, e.g. gutters with a slippery surface (see Figure 4) or tramways, especially in bad weather conditions.

Children did not like unevenness in the cycle paths, because these vibrations may damage their bicycle or make them fall and hurt themselves. Parents were also concerned about the fact that their child may not have seen these holes and therefore, the child may be surprised and fall.

*“ … but in the other street I am concerned about the holes in the road. The children are not allowed to cycle to school only for that reason. You must cycle in the middle of the street to be actually able to cycle, even with the car you do not drive through those wells. The day that street is repaired, the children will cycle to school.” (Mother of girl, 36.5 yrs)*

#### Legibility of road situations

Parents found a lack of legible road line markings a major issue that makes it unclear where cyclists have to ride. Making cycling facilities clearly visible and understandable by road signatures or colors were elements suggested by children and parents to facilitate legibility. For example, the sudden disappearance of road markings of the cycle path was disliked.

*“Suddenly, the cycle path stops, or at least the road markings. The best option would be crossing the street, since here is no cycle path anymore …. So it is very unclear where to cycle. Instead of suddenly stopping the road markings, they should have made a diagonally road marking to the other side so that you knew where to cycle.” (Father of a boy, 41.4 yrs)*

#### Crossings

**Figure 5:**
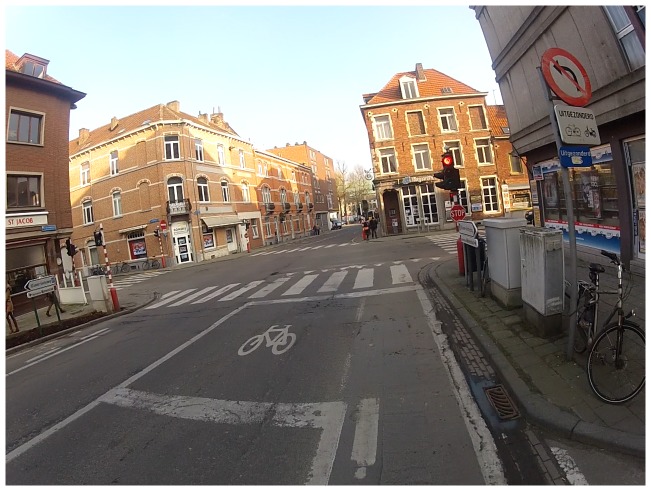
Participants liked bike boxes to cross roads, since they make cyclists visible before crossing roads.

Parents disliked their child having to cross roads, especially when it was unclear where cyclists needed to cross. Designated places to cross, such as crosswalks or bike boxes (see Figure 5), were therefore viewed favorably, since these infrastructures made cars alert of the presence of cyclists. The presence of traffic lights was considered a good way to create clarity about when cyclists may cross the road. However, parents felt that traffic lights should be properly adjusted so that it is clear to all road users who may cross. For example, different traffic lights for pedestrians and cyclists can make it unclear to cars when cyclists are allowed to cross.

#### Continuity of cycle facilities

Both parents and children indicated that a good cycle network is preferable for cycling for transport. Children found it less enjoyable when they often had to get off their bicycle such as needing to press the traffic lights button or wait at zebra crossings.

### Road design

#### Obstacles

Road constructions, incorrectly parked cars, animals, poorly maintained hedges or blown branches were mentioned as obstacles, making it more difficult to cycle for transport.

#### Intersections

Crossings and roundabouts were mentioned by parents as difficult traffic situations which are not always understood by children. Children found it difficult to maintain an overview when cars are coming from different streets. Some parents said that it is easier for their child to cross by dismounting their bicycle and walking. Other parents mentioned that their child is able to handle these difficulties, but the child just needs to be very alert. Bicycle tunnels and traffic lights were seen as good solutions to avoid difficulties in crossing junctions or roundabouts.

*“This is difficult because there are several streets that come together. It is difficult because you have to watch the traffic. And if you have seen the latter, still a new car can arrive. I am then worried that the car would hit me, which probably will not happen, but still …” (Boy, 11.7 yrs)*

#### Traffic signs

Parents were concerned about their children being not aware of the traffic rules and signs while cycling. Some children said that they did not pay attention to traffic signs, while other children indicated that they liked traffic signs because they make situations more understandable.

### Facilities at destination

Parents cited that their child is not allowed to cycle for transport if no secure storage was present at the final destination because of fear of bicycle theft.

### Aesthetics

Children indicated that places that were aesthetically appealing (e.g. natural elements, historical buildings, clean and quiet streets, open spaces) were also more inviting to cycle for transport. Parents did pay attention to aesthetics, but these were linked with safety concerns. For example, parents indicated that nice buildings or parks were inviting to cycle along, but they are concerned that this would distract their children from the focus on traffic. Or if there is a lot of noise, children are not able to hear cars driving behind them, making it less safe to cycle.

*“Ah, the city park, nicely quiet, beautiful images, great fun to ride, you will see other things than you are used to and that is sometimes nice to watch. It is nice to cycle here, because you can also see people running, or playing football or basketball, very nice to see.” (Girl, 11.4 yrs)*

*“I find it a quite nice street. Most streets are so … grey, but with that school, there is some color in the street.” (Girl, 10.2 yrs)*

### Topography

Both parents and children agreed that steep inclines are less comfortable to cycle because of the physical effort. Parents indicated that cycling downhill increases the speed of cycling, and therefore, children have less time to observe traffic situations.

### Weather

#### Amount of daylight

Parents and children did not like to cycle during evenings when it was too dark. Parents said that children are allowed to cycle more during summertime compared to the winter because of the amount of daylight. Street lightning was mentioned as essential to cycle in the evenings, due to parents' fears of stranger danger and the limited visibility of children.

#### Amount of precipitation

Parents said that on rainy days, they were more likely to chauffeur their child to the destination than letting their child cycle through the rain.

### Social control

Parents indicated that they were less likely to let their child cycle on abandoned streets. In case of an accident, no one would be able to help their child. Children and parents liked cycling within a group although the latter were concerned of their child paying more attention to their friends instead of to the road.

### Stranger danger

Parents and children were concerned about the presence of immigrants, homeless persons, drunk people, groups of youth or other people they do not know. Some parents were afraid of their child being kidnapped or bullied on the road. Children indicated that they did not like to pass groups of youth or mentioned avoiding places where they could meet drunk people, for example, bicycle tunnels or bushes.

### Familiar environment

Both children and parents mentioned that cycling in an environment which the child already knows was more comfortable compared to a new environment. Parents stated that their child knows the difficulties in this familiar environment and, therefore, parents were less worried about their child being in dangerous situations. For example, a girl explains: *“I think it is also nice here because I already know it here. If you go somewhere where you do not know the place, it is like ‘what is going to happen here?’ and ‘which way we have to go now?’ But here you know your way … I like it because it is always the same ritual.” (Girl, 11.5 yrs)*

## Discussion

The objective of the current study was to improve our understandings of environmental factors potentially influencing cycling for transport to various destinations in Flemish 10- to 12-year old children. We used bike-along interviews with child and parent pairs to obtain context-specific and detailed factors influencing children's cycling for transport. Although cycling was the most preferred transport mode for children in the current study, motorized transport was still the major transport mode to school in this small sample. This discrepancy between the preferred and the actual transport mode suggests that cycling for transport was popular among these children in their last years of primary school, but parents restricted their children from cycling. The main reason for parents not allowing their children to cycle was because they perceive traffic situations as too dangerous. This is consistent with previous research [23], [25], [48], but the current study adds several other environmental factors related to traffic safety and cycling which have not been examined in other studies.

In the current study, participants highlighted the importance of certain physical characteristics of cycle paths. The ideal cycle facility was described as wide and well separated from traffic by having an independent cycle track or having a cycle track next to the road, but secluded by a shoulder or a hedge. Additionally, the type of surface and evenness of the cycle path was mentioned as being important in order to cycle comfortably. These segregated, wide and even cycle facilities should be accompanied by proper intersection treatment. These physical characteristics could be used in future studies identifying the most efficient cycle facilities to increase children's willingness to cycle for daily travel [49], [50], [51].

Traffic situations should be made understandable and legible in order that children do not have to worry about where to cycle or where to cross, so that they can focus on oncoming traffic. Bike boxes (see Figure 5) put cyclists in front of cars, which makes cyclists visible in traffic and indicates where they should cross. Bike boxes also help cyclists avoid inhaling exhaustion fumes from motor vehicles. Constructing a single traffic light for both pedestrians and cyclists may also be an effective strategy for ensuring clear traffic situations are established. Urban planners should take into account that children cycle at a lower height and are less visible for other road users and have a more limited view of the traffic situation compared with adults. Therefore, cars should not be allowed to park at intersections and obstructing vegetation should be removed. On the other hand, children should make themselves more visible by wearing fluorescent items. Parents and schools should encourage children to be more visible in traffic, especially during the darkest moments of the day. Increasing children's knowledge of traffic signs, perhaps through road safety education in schools, is another recommendation for increasing children's traffic safety.

Future studies should investigate whether increased knowledge of road and traffic safety among children would increase parental trust, and therefore lead to an increase in children's cycling. Parents were less concerned about their child cycling alone if they had to navigate challenging routes that were familiar to them. Knowledge of a particular environment can be achieved by repeatedly cycling the same route, in order to learn where road dangers are situated. Therefore, parents should be encouraged to accompany younger children on the route to various destinations while indicating traffic difficulties. This way, when children get older, they will know the difficulties on these routes and they should be able to deal with traffic situations. Parents should also be encouraged to choose the safest route for their child so that this route becomes a habit and increases the chance of becoming the preferential route in the future.

Next to these new, detailed and context specific variables, participants highlighted the importance of various environmental factors which were previously studied in relation with cycling for transport [22], [23], [25], [27], [28], [29], [31]. Traffic density, traffic speed, presence of intersections, presence of cycle facilities, distance to destination, weather, amount of inclination, amount of roads to cross, personal safety, aesthetics and connectivity were mentioned as being associated with cycling for transport in children in the current study. When parents were asked which environmental factors were most likely to influence them to allow their child cycle for transport, traffic safety was almost always cited as the most important barrier. This finding was not surprising, as the child's risk to get (seriously) injured or killed during cycling is in Flanders still much higher than while being a passenger in a car [43]. These actual cycling risks are perceived by parents, but might be susceptible to individual differences which are attributed to parent's own (cycling or traffic) experiences, attitudes and beliefs [52]. In the current study, parental perceived safety is considered as a main correlate of transportation cycling. Individual differences in perceptions of cycling risks may be a stronger predictor than the actual risk for cycling [32]. Future research should determine if increases in objective traffic safety leads to changes in parental perceived safety and a higher share of transportation cycling among children. Parents were very concerned about their child's safety en route to a destination, which was often influenced by certain environmental factors. For example, high connectivity was preferred by parents because it ensures the possibility of choosing the safest route for their child to cycle along. Aesthetics were also mentioned as being important by both parents and children, but parents were concerned of the potential distraction of these elements from focussing on traffic. It can therefore be concluded that the environment should evoke feelings of safe cycle routes, especially in parents. These perceptions, rather than objective cycling risks, are considered to be a correlate of the frequency of cycling for transport among children. This is consistent with a review on personal and traffic safety in children [30]. The authors concluded that it is parental perception, rather than children's perceptions of road safety, which is related to independent mobility of children [30]. This indicates that, even at this age, parents are the major decision makers in transport related choices.

Our findings also indicated that environmental factors might interact with each other. The effect of one environmental factor may be strengthened or inhibited by the presence or absence of any other variable. For example, the presence of cycle facilities was reported to be more important in streets with high traffic speed and a large amount of traffic passing. Furthermore, findings suggest that the presence of one factor can have multiple (both positive or negative) effects on cycling for transport. For example, bicycle tunnels were seen as a good alternative to avoid crossing at busy intersections, but these were also reported as places where drunk persons or other strangers may be present. Also, the presence of natural elements (e.g. trees, hedges) were liked by the participants, but poor maintenance of these greeneries can obstruct the view on oncoming traffic. These are just some examples indicating the need to study the relative importance of various environmental factors. It is certainly also important to study the effect of combinations of factors, since these are also present in real life situations. Recently, two quantitative studies showed the existences of these interactions between environmental factors related to transportation walking in older adults [53], [54].

The current study has several strengths. It used bike-along interviews to identify context-specific and detailed information on factors influencing children's cycling for transport. The objective environment was assessed by the video recordings which were accompanied by the subjective perceptions of the environment. This ensures that no recall bias could occur and rich and detailed information was obtained [36], [37]. While cycling in the target environment, real life combinations of factors were present and, therefore, bike-along interviews allowed the participants to discuss the presence of several environmental factors at the same time with the researchers. The method allowed us to obtain new and very rich data (e.g. visibility, properties of cycle facilities, familiarity of the environment etc.).

Some limitations of this study should be considered. Most participating parents had a high socio-economic status (74% had a higher education degree). Additionally, most participants were girls (66%) and mothers (80%), which may have affected our results. Women are more concerned about safety issues [55], and it is possible that this may have overestimated the importance of road safety to cycle for transport in children. However, during the bike-along interviews, no apparent gender differences related to safety concerns were observed by the researchers. Although both regular and non-regular cyclists and urban and semi-urban inhabitants were included, it was outside the scope of this paper to investigate differences in perceptions between these groups. Future studies need to identify if the association between transport-related physical activity and the environment is similar among different subgroups of children [56]. Furthermore, the findings are only generalizable for (semi-)urban areas of Flanders, which limits generalization to rural areas and to other countries. Finally, parents indicated that environmental factors are important, and that the child's individual characteristics were also at least as important. For example, parental perceptions of their child's cycling skills were mentioned by parents to influence their decision regarding whether their child was allowed to cycle for transport. A previous study has shown that a cycle skills training of only three lessons was effective for improving children's cycle skills [57]. Therefore, parents and schools should be encouraged to promote cycling skills of primary school children with education programs.

## Conclusions

Bike-along interviews were able to identify new, context-specific environmental factors that potentially influence cycling for transport in 10- to 12- year old children. Moreover, the study confirmed the existence of associations between previously studied environmental factors. Our findings suggest that, to increase the amount of cycling in this age group, parental perceptions of traffic safety is the main issue that should be addressed. Therefore, the environment should provide wide and even cycle tracks. Intersections should be free of parked cars and hedges to allow a good overview of the traffic situation. Traffic density and speed should be lowered by installations of speed bumps and chicanes. Finally, parents should be encouraged to cycle with their children to various destinations from a young age, so that 10- to 12-year old children know the difficult traffic points and are able to cycle alone at an older age. Experimental studies should investigate whether changes in these environmental factors would lead to changes in levels of children's transportation cycling.
